# An introduction to spatial transcriptomics for biomedical research

**DOI:** 10.1186/s13073-022-01075-1

**Published:** 2022-06-27

**Authors:** Cameron G. Williams, Hyun Jae Lee, Takahiro Asatsuma, Roser Vento-Tormo, Ashraful Haque

**Affiliations:** 1 Department of Microbiology and Immunology, University of Melbourne, located at the Peter Doherty Institute for Infection and Immunity, Parkville, VIC 3000 Australia; 2 Cellular Genetics Group, Wellcome Sanger Institute, Wellcome Genome Campus, Hinxton, Cambridge, CB10 1SA UK

## Abstract

Single-cell transcriptomics (scRNA-seq) has become essential for biomedical research over the past decade, particularly in developmental biology, cancer, immunology, and neuroscience. Most commercially available scRNA-seq protocols require cells to be recovered intact and viable from tissue. This has precluded many cell types from study and largely destroys the spatial context that could otherwise inform analyses of cell identity and function. An increasing number of commercially available platforms now facilitate spatially resolved, high-dimensional assessment of gene transcription, known as ‘spatial transcriptomics’. Here, we introduce different classes of method, which either record the locations of hybridized mRNA molecules in tissue, image the positions of cells themselves prior to assessment, or employ spatial arrays of mRNA probes of pre-determined location. We review sizes of tissue area that can be assessed, their spatial resolution, and the number and types of genes that can be profiled. We discuss if tissue preservation influences choice of platform, and provide guidance on whether specific platforms may be better suited to discovery screens or hypothesis testing. Finally, we introduce bioinformatic methods for analysing spatial transcriptomic data, including pre-processing, integration with existing scRNA-seq data, and inference of cell-cell interactions. Spatial -omics methods are already improving our understanding of human tissues in research, diagnostic, and therapeutic settings. To build upon these recent advancements, we provide entry-level guidance for those seeking to employ spatial transcriptomics in their own biomedical research.

## Background

### Why study ‘cells’ with ‘-omic’ detail?

As an early pioneer of the microscope, Robert Hooke coined the term ‘cell’ in the 1600s to describe structures observed under magnification in plant material; ~ 400 years later, the word and the concept has stuck, achieving primacy as a fundamental unit of life on Earth. Unicellular organisms epitomize how cells are governed by their nucleic acids, by their DNA, which houses ‘genes’ that encode proteins, and messenger RNAs (mRNA), which act as an intermediary between the two. This is also true for multi-cellular life, including human beings. Indeed, our understanding of ‘cell theory’ underpins almost every aspect of human health and disease, from islet cells in the pancreas protecting against diabetes, immune cells making protective antibodies against bacteria and viruses, or neuronal cells storing life experiences and memories. A comprehensive understanding of how individual cells employ their mRNA and proteins in different tissues of the human body may lead to new strategies to prevent or treat infections, cancers, neurological or metabolic disorders, and a plethora of other conditions. Thus, the ongoing challenge is to examine individual cells at genome-scale, and with molecular detail.

### A decade of studying mRNA in single cells

It is universally accepted, although no less remarkable for it, that the hundreds of types of cells that comprise the human body possess essentially the same DNA. Thus, cellular diversity and cell-specific function, initiated during embryogenesis and perpetuated throughout adult life, is best assessed not at the DNA level, but at the protein level. However, there are no commercially available methods for quantifying the thousands of proteins within individual cells of our bodies. Instead, in 2009 came the first description of the entire repertoire of mRNA (up to ~ 20,000 genes) from a single cell, known as the transcriptome [1]. This triggered an explosion in efforts to study heterogeneity and dynamic change in eukaryotic cells using single-cell transcriptomics, more commonly referred to as single-cell RNA-seq (scRNA-seq) [2].

Most scRNA-seq methods have required single cells to be released intact and viable from tissues, which are then assessed and/or labelled individually using microfluidic, droplet-based, or limiting dilution methods—a notable exception is single-nucleus RNA-seq, in which nuclei from tissues are mechanically recovered and processed [3]. The (current) most common commercially available platform, the Chromium Controller from 10x Genomics, isolates single cells or nuclei in droplets in an oil-based emulsion, where mRNA capture, reverse transcription, and molecular and cellular barcoding is conducted. This technology has permitted unbiased, genome-scale assessments of cellular identity, heterogeneity, and dynamic change for thousands to hundreds of thousands of cells. Continued scaling of such projects means it will be commonplace to study millions of cells, as computational and financial challenges are navigated [4].

### What is ‘spatial transcriptomics’ and why is it useful?

Despite the ongoing success of scRNA-seq, a crucial practical obstacle exists: the need to liberate viable cells from whole tissue without inducing stress, cell death, and/or cell aggregation. Immunologists have perhaps benefitted the most from scRNA-seq because many immune cells (particularly T and B lymphocytes) are not always anchored in tissues and are therefore relatively easy to isolate from circulating blood, lymphoid organs, peripheral tissue, and even tumours [2, 5–7]. In contrast, many other cell types, e.g. neurons in the brain, remain less amenable to scRNA-seq analysis, requiring specialized tissue dissociation protocols to recover them [3, 8]. Therefore, purely from a technological standpoint, there has been an impetus to conduct transcriptomics on intact tissue. Since spatial information is preserved by studying intact tissue, these methods have been referred to as spatially-resolved transcriptomics, or simply ‘spatial transcriptomics’.

The position of any given cell, relative to its neighbours and non-cellular structures, can provide helpful information for defining cellular phenotype, cell state, and ultimately cell and tissue function. Location can determine the signals to which cells are exposed. While endocrine signals act at macroscopic scales, many other types of signals act upon neighbouring cells via cell-cell interactions or via soluble signals acting in the vicinity. One form these signals can take is of cell surface-bound protein receptors and ligand pairs, the mRNA for which can be detected by transcriptomics [9]. Therefore, a prime driver for rapid developments in spatial transcriptomics is the assertion that tissue context aids assessment of cell biology, which has been true for 2-photon intravital imaging of immune cells in tissues [10], and will likely also apply to transcriptomes within tissue.

Furthermore, it is becoming increasingly apparent that sub-cellular localization of mRNAs varies according to gene function, regulating for example where a protein product is produced and trafficked in cells [11]. This is a common phenomenon, affecting an estimated 70% of transcript species [11, 12]. In past decades, these inferences were made by targeted screens of specific mRNAs but are beyond the current capabilities of scRNA-seq. Emerging spatial transcriptomics techniques promise to profile simultaneously hundreds to thousands of genes at subcellular resolution.

Although technologies for counting and profiling transcripts in tissue have existed for decades, it was only in 2021 that spatial transcriptomics was named ‘Method of the Year 2020’ by *Nature Methods* [13]. The field continues to grow fast, driven by numerous factors including the reduced cost of next-generation sequencing (NGS), initiatives such as the Human Cell Atlas (HCA), and the BRAIN Initiative Cell Census Consortium (BICCC) [14, 15], increases in computing capacity, and improvements in microscopy and imaging. During this growth phase, wet-lab technologies and computational approaches for generating and analysing spatially-resolved transcriptomic data are rapidly evolving and improving, as was the case for scRNA-seq from 2009. While scRNA-seq techniques have seen widespread uptake, much of the current literature on spatial transcriptomics is technical, and not oriented to researchers unfamiliar with the field. Here, we draw on spatial transcriptomics literature and several recent reviews [16–19] to provide an introductory guide to spatial transcriptomics, covering available techniques, experimental design considerations, and bioinformatic analyses needed to reveal novel biology. In particular, we provide a summary of existing spatial transcriptomics techniques and literature, and a framework for researchers to: 1. determine an experimental design shaped by sample availability and specific features of biological systems being studied; 2. select the best-suited spatial transcriptomics technique; 3. maximize output from spatial transcriptomic studies using complementary data such as single-cell transcriptome reference data and auxiliary stains; and 4. analyse these data with specialized algorithms for spatial -omics.

## Spatial transcriptomics in current biomedical research

Spatial transcriptomic techniques have existed for almost a decade, but until recently only at the institutes where they were developed. Commercialized techniques such as *Spatial Transcriptomics* [20], released as *Visium* by 10X Genomics, as well as *GeoMx* [21] and *CosMx* [22] by Nanostring, have made spatial transcriptomics more accessible. Other -omics techniques such as ‘spatial proteomics’ (a term currently employed to describe targeted assessment of tens of proteins via antibody-based methods), spatial assays for chromatin accessibility, and spatial genomics have also contributed recent findings [23–27]. Here we showcase recent applications of spatial -omics, with a focus on transcriptomics, to elucidate, for example, broad patterns of gene expression in tissue differentiation, spatially localized disease mechanisms, and specific cell types driving disease, in cancer, neuroscience, and reproductive biology.

At a broad level, spatial transcriptomics can be used to characterize transcriptional patterning and regulation in tissues. For example, in reproductive biology, one goal has been to define mechanisms regulating cellular differentiation in the endometrium, the mucosal layer of the uterus, as it grows and is shed at menstruation [28]. Applying *Visium* and scRNA-seq to human endometrial samples revealed patterns of gene expression in healthy tissues such as gradients of WNT and NOTCH pathways in different epithelial cell states located at specific regions of the tissue. Large field-of-view imaging can also reveal new structural features in diseased tissues. Recent spatial proteomic studies, targeting 40-50 proteins in human tumours, revealed gradients of histological features such as grade and morphology that coincided with protein gradients in colorectal cancer [29], and immunological correlates of complete response to therapy in HER2^+^ breast cancer [30]. Spatial -omics techniques have also revealed genomic features in healthy and diseased tissue, for example the spatial distribution of cancer clones in mouse models of metastasis and primary human cancer [26]. Thus, spatial -omics techniques can improve our understanding of tissue architecture and its molecular underpinning in health and disease.

At a finer level, spatial transcriptomics can reveal tissue neighbourhoods and local features contributing to disease. In neuroscience, spatial transcriptomics’s advantages are two-fold. Firstly, it removes the need for tissue dissociation of delicate neurons. Secondly, it preserves the spatial context of cells. One study employed *Spatial Transcriptomics* to determine gene modules expressed in the local vicinity of amyloid plaques in murine Alzheimer’s disease model [31]. Using spatial transcriptomics, and contrary to earlier reports, this study suggested that proximity to amyloid plaques induced gene expression programs for inflammation, endocytosis, and lysosomal degradation [31]. They also observed changes specific to oligodendrocytes, including upregulated myelination genes. Finally, these transcriptomic changes were also observed in human tissue using a different targeted, high resolution spatial transcriptomics technique, *in situ sequencing (ISS)*. In this case, spatial context revealed differential regulation of immune genes, particularly complement genes in the vicinity of amyloid plaques, suggesting a novel disease mechanism. A study of primary cutaneous melanoma used high-plex, subcellular-resolved, fluorescent protein imaging via *CyCIF* [25] to identify molecular programs associated with histopathologic progression [32]. This revealed highly localized immunosuppressive niches containing PDL1-expressing myeloid cells in contact with PD1-expressing T cells, often juxtaposed by areas of T cell immunoediting. Finally, a recent study of distal regions of the healthy human lung discovered new types of fibroblast, alveolar, and secretory cells [33]. Thus, spatial technologies are providing new information on specific tissue niches in health and disease.

Spatial transcriptomics has been used to study in detail individual cells and cell types. For example, a recent human breast cancer study [34] used an unbiased (i.e. characterizing expression of every gene in the genome) technique, *Visium*, as well as droplet-based scRNA-seq to profile 26 cancer sections of various clinical subtypes including ER^+^ and HER2^*+*^. The authors computationally identified tissue regions representing tumour cell, stromal, and immune cell regions solely from gene expression profiles. The regions agreed with pathologist annotations, but interestingly did not require manual intervention for annotation; in doing so this provided detailed expression profiles for thousands of genes. Second, spatial analysis based on a droplet-based scRNA-seq reference, revealed PD-L1 and PD-L2-expressing macrophages enriched in CD4^+^ and CD8^+^ T cell areas, which was associated with poor survival in a large patient dataset. Others have used highly-resolved multiplexed imaging techniques to probe expression of pathways in individual cells, for example, a glioblastoma study used *CyCIF* to identify CD73-expressing tumour cells colocalizing with microglial cells that expressed CD39 [35]. These two molecules act in a purinergic signaling pathway to support tumour proliferation and invasion, with this spatial colocalization associated with poorer clinical outcome [35].

Overall, spatial transcriptomics is broadly applicable to human and mouse tissues in both steady state and disease. Spatial techniques can reveal features from tissue-wide patterning to disease-promoting cell niches and even single-cell biology. Until recently, only a few commercial spatial transcriptomics platforms have existed, *e.g. Visium* from 10x Genomics, and *GeoMx* from Nanostring [21], or high-resolution, targeted methods like *RNAscope* [36]. Increasingly, a wider array of technologies with diverse technical foundations and research capabilities are entering the market, which will be discussed in the next section.

## Spatial transcriptomic technologies

Spatial transcriptomics aims to count the number of transcripts of a gene at distinct spatial locations in a tissue. Different techniques have different technical parameters. The tissue size can vary from a small (<1mm^2^) section to whole organ sections from model organisms; the number of genes counted can vary from tens to thousands or even the whole genome; a spatial location may range from a whole tissue domain, to a large 500 μm × 500 μm region of interest, down to a single cell or even finer. With current technologies, there is often a trade-off between the number of genes profiled and the *efficiency* of the technique—the proportion of transcripts of interest that are successfully counted, ranging from near 100% to as low as 1% [17]. Here, we review different methods for conducting spatial transcriptomics and their technical parameters.

**Fig. 1:**
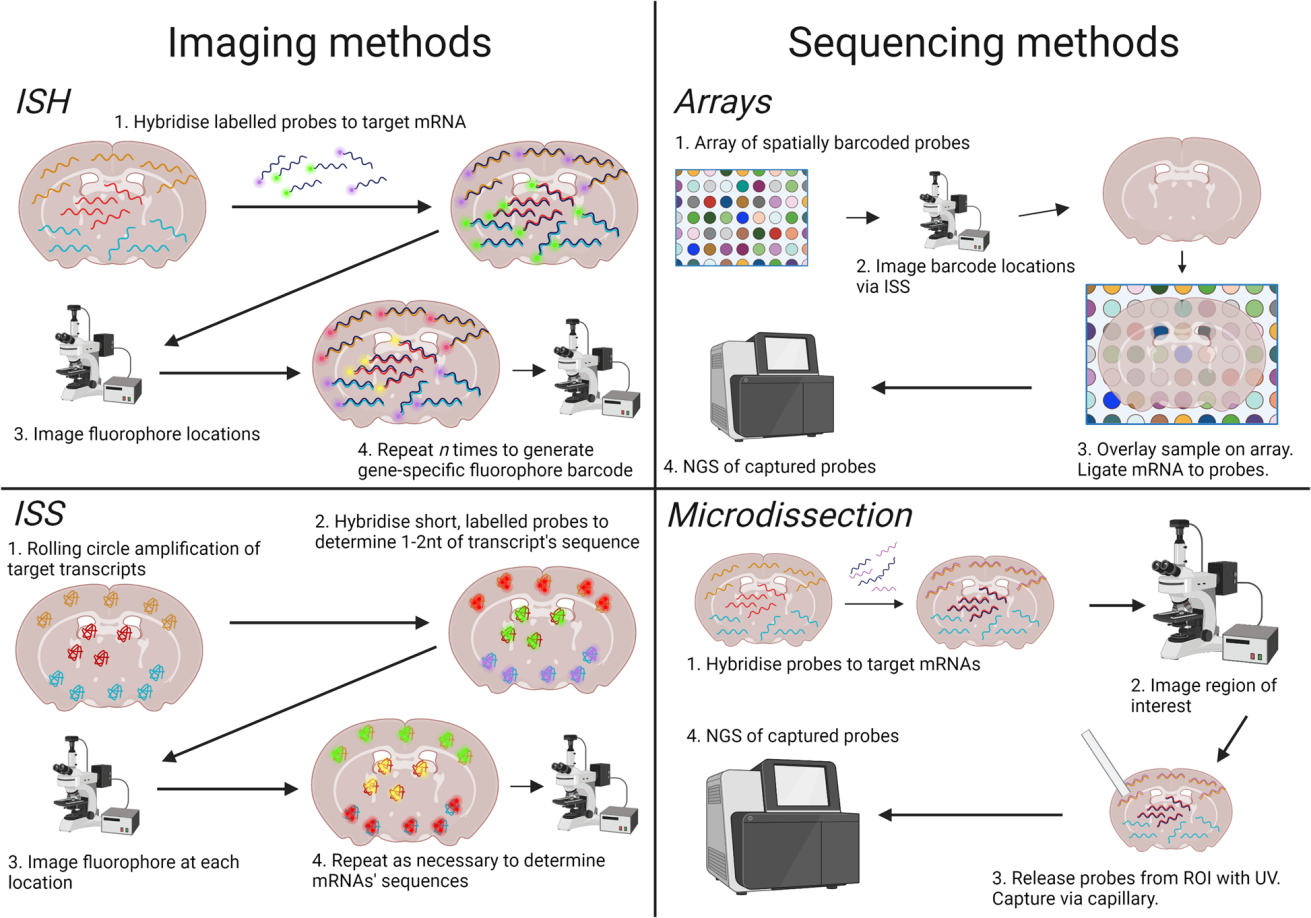
Four different ways to record location and species of mRNA transcripts. Transcripts can be imaged directly in intact tissue by hybridization to fluorophore-labelled probes, or their locations can be recorded before they are extracted and undergo NGS. Transcript species can be imaged repeatedly with the same probes but different fluorophores to create a gene-specific barcode as in ISH. Short probes can also be imaged to read along an amplified transcript and determine its sequence as in ISS. Arrays of spatially barcoded probes can be used to label mRNAs with a sequence indicating location before undergoing NGS. Finally, cells or regions of interest can be directly microdissected and their locations recorded before their transcriptomes undergo NGS. Created with biorender.com

Broadly, recent reviews [16–18] propose that there are two ways to profile transcriptomes while preserving spatial information; firstly, by imaging mRNAs *in situ* via microscopy (Fig. 1). This is the foundation of *imaging-based spatial transcriptomics technologies.* When imaging mRNAs *in situ* there must also be a means of distinguishing different mRNA species, of which there are two [17]. One is *hybridization* of mRNAs to fluorescently labelled, gene-specific probes. Hybridization refers to polymerization of single-stranded mRNAs to single-stranded probes with a complementary sequence. This spatial transcriptomics technique is called *in situ* hybridization (ISH). The other is *in situ* sequencing (ISS) of amplified mRNAs, in which transcripts are directly sequenced inside a tissue block or section by sequencing by ligation (SBL) technology, discussed below. Among imaging-based technologies we therefore highlight *in situ hybridization (ISH)-based methods* and *in situ sequencing (ISS)-based methods* (Fig. 1).

Secondly, the other broad method of spatial transcriptomics is to extract mRNAs from the tissue while preserving spatial information and subsequently profile mRNA species via next-generation sequencing (NGS) techniques (Fig. 1). This is the foundation of *sequencing-based spatial transcriptomics technologies* (*sequencing* referring to NGS rather than ISS)*.* Common methods of preserving spatial information are (1) via direct capture and recording of location, such as via microdissection and microfluidics, and (2) via ligation of mRNAs to spatially-barcoded probes in a microarray [17]. Hence, among sequencing-based technologies we highlight *array-based methods* and *microdissection-based methods* (Fig. 1).

Below, we review published spatial transcriptomics techniques regardless of commercial availability to outline and compare technologies, advantages, disadvantages, and applications.

### Imaging-based technologies

Imaging-based spatial transcriptomics use in situ hybridization of mRNAs with labelled complementary probes, detected by microscopy to quantify transcripts. A recent review [17] identifies early steps towards these technologies as beginning in the 1960s, with labelling of nucleic acids via complementary probes in 1969 [37, 38], labelling of specific sequences in 1973 [39], and fluorescent-labelling in the late 70s [40, 41]. This in situ hybridization (ISH) was first used on a whole organism (*Drosophila*) in 1989 [17, 42]. ISH was useful for profiling gene expression patterns in tissue sections and whole organisms but was largely qualitative. A quantitative method for imaging probe-labelled transcripts emerged in 1998, single-molecule fluorescence in situ hybridization (smFISH), in which each labelled transcript appears as a single spot via microscopy [43]. Variations of this have been commercially available for some time, such as *RNAscope* [36]. Modern imaging-based spatial transcriptomics is founded on smFISH. As in recent reviews [17, 18], we specify below two categories of imaging-based spatial transcriptomics: ISH-based, and ISS-based.

Current ISH methods include *seqFISH* [44], *seqFISH*+ [8], and *MERFISH* [45]. All three extend upon smFISH by employing multiple rounds of hybridization. Instead of 1:1 fluorophore to gene correspondence, which is impossible for thousands of genes, *seqFISH* (sequential fluorescence in situ hybridization) uses a temporal barcode of fluorophores in which the same probes are hybridized in different *rounds* of hybridization, but each time with a different fluorophore. The combination and order of fluorophores is specific to a gene. In contrast, *seqFISH*+ uses primary gene-specific hybridization probes, each with multiple binding sites to hybridise secondary fluorophore-labelled probes. In each successive round of hybridization, the next binding site is hybridized to a fluorescent probe and the whole sample is imaged, with the sequence of fluorophores defining the mRNA species. *seqFISH* uses 4 fluorophores, with barcode length *n* depending on the number of genes targeted, to profile up to the whole genome in vitro [44]. However, it is limited in practice by optical crowding if too many transcripts are profiled [8]. *seqFISH*+ can profile 10,000 genes in a single cell using, instead of 4-5 fluorophores, 60 ‘pseudocolours’ and labelling only a fraction of transcripts with a fluorophore at each hybridization round, avoiding optical crowding [8]. Each pseudocolour is derived from a combination of 20 individual hybridization rounds. *MERFISH* (multiplexed error-robust fluorescence in situ hybridization) also uses sequential hybridization, but instead of a sequence of fluorophores uses a sequence of sites for binary-encoded secondary probes: fluorophore-labelled or unlabelled [45]. Each hybridization round in any imaging technique is subject to a risk of error; the risk of an error for any transcript grows exponentially with each round [45]. *MERFISH*’s binary approach is robust to error as it reduces the chance of an irreconcilable error from one round preventing identification of a transcript, because if an unexpected sequence is determined, it can more easily be corrected to an expected sequence than if 4 fluorophores were used. Other than error, two limitations noted for some ISH-methods are the small size of tissue profiled [46], ~1mm^2^ for *seqFISH+*, and time required to repeatedly image*.* A recent technique, enhanced electric FISH (*EEL FISH*), electrophoretically transfers mRNAs from tissue onto glass coverslips prior to *FISH* [46], which condenses tissue depth (*z*-axis), allowing greater signal strength for images captured in the x/y plane and reducing imaging time. This is undergoing commercialization as Rebus Biosystems’s *Esper* instrument. Finally, *EASI-FISH*, or expansion-assisted iterative fluorescence ISH, which uses hydrogel expansion of cleared tissue [47] to clear thick tissue, blocks up to 300 microns thick, before using a *seqFISH*-like encoding strategy of direct probe hybridization to target mRNAs and imaging [48]. Thus, the technique offers 3D resolution of gene expression in tissue. Recent commercial techniques for 3D resolution of gene expression in tissue include Nanostring’s *CosMx* ISH-based instrument [22].

ISS methods, instead of using gene-specific probes, use probes to profile 1–2 bases at a time of primed and amplified transcripts in the tissue. Each base or 2 base sequence is linked to a different fluorophore, enabling visualization and recording that leads ultimately to the identification of each transcript. Primers may be targeted or untargeted, with amplification usually via rolling circle amplification (RCA), which preserves spatial localization. The first example of this for spatial transcriptomics was released in 2013, before being commercialized as *Cartana* [49], later purchased by 10X Genomics. *Cartana*, a targeted method, used one query base per round, with later untargeted techniques, e.g. fluorescence in situ sequencing (*FISSEQ*) and *ExSeq* (a combination of *FISSEQ* with expansion microscopy) using two bases per round [50, 51]. Finally, *STARmap* extends methods to 3D tissue blocks and employs error-robust sequencing with error-reduction by dynamic annealing and ligation (*SEDAL*) with a combination of 1 and 2 query base probes [52]. However, imaging in the *z*-axis may require long microscopy times [46], particularly if multiple rounds of *STARmap* to profile different panels of genes in the same block are performed.

ISH and ISS methods are useful due to their high spatial resolution, which is capable of profiling mRNA at subcellular level. ISH methods detect mRNA more efficiently than ISS methods because rolling circle amplification can be selective [17]. However, ISS methods can examine larger tissue areas because RCA increases signal-to-noise permitting lower magnification. Both ISH and ISS-methods suffer some similar technical limitations, in particular a requirement for many hours to days of imaging time on a microscope, thus generating terabytes of data. Finally, these methods require some trade-off between capture efficiency vs number of genes profiled. ISH methods such as *seqFISH can* count almost all target transcripts in a sample, but the more genes are profiled, the more rounds of hybridization are required and the greater the potential for compounded errors. Conversely, ISS methods such as *Cartana* require lower magnification settings, but RCA is inefficient and non-amplified transcripts are not counted, meaning capture efficiency is comparable to sequencing methods discussed below [17, 18].

### Sequencing-based technologies

Sequencing technologies capture mRNAs from the tissue, synthesize cDNAs, then count gene-specific sequences via NGS. Importantly, positional information is retained at the point of mRNA capture. An early pre-cursor technology was laser capture microdissection (LCM), in which specific tissue regions were processed for transcriptomic profiling via microarrays [17]. This was followed in the 2000s by *Tomo-seq*, in which tissue was cryosectioned, with each section undergoing RNA-seq profiling [53]. *Geo-seq* is similar, but tissue sections are subjected to scRNA-seq [54]. Modern microdissection techniques include Nanostring’s *GeoMx* DSP [21] with variable spatial resolution down to near single cells, in which genes are barcoded with gene-specific photocleavable probes. When UV light is shone on the tissue, one region of interest at a time, probes are released and sequenced. Due to laser-induced mRNA degradation during LCM, and practical considerations such as the number of independent library preparations, *STRP-seq* was developed to profile gene expression in 2D across consecutive thin sections that are then cross-sectioned at different angles and sequenced to reveal gene expression patterns [55]. Overall, these microdissection-based techniques provide a useful first set of techniques for profiling unbiased, spatially resolved transcriptomes, but they are limited by their spatial resolution, degradation of mRNAs when LCM is used for microdissection, and by the need to process many samples for sequencing [17].

In contrast, an early example of positionally-barcoded, ‘array-based’ capture of mRNA was *spatial transcriptomics* (*ST*) in 2016 [20]. Tissue was mounted over an array, such that released mRNA was captured locally by spatially-barcoded probes, converted to cDNA, and then sequenced. Probes were not gene-specific, as with ISH and some ISS methods, instead capturing polyadenylated mRNA in an untargeted manner. The spatial resolution of array methods is defined by the size of a capture area harbouring any given unique positional barcode, analogous perhaps to pixel size in photography. *ST* had 100 μm (centre-to-centre) capture areas, or pixels [20]. Its commercialization by 10X Genomics, termed *Visium*, improved this to hexagonal 55 μm resolution, with plans for 2 μm capture areas in 2022. Other developments included *Slide-seq*, which used arrays composed of 10 μm-diameter barcoded beads, with barcodes at each location determined prior to tissue mounting [56]. A version with improved barcoding and enzymatic library preparation, *Slide-seqV2*, recovers ~ 30–50% as much transcriptomic information per capture bead as droplet-based single-cell transcriptomics from 10X Genomics, meaning that hundreds or thousands of genes can be detected per 10 μm-pixel [57]. *High-definition spatial transcriptomics (HDST)* works similarly to *Slide-seqV2*, with beads confined to wells etched in the slide and a spatial resolution of 2 μm [58]. Emerging technologies such as *Stereo-seq* have achieved even lower resolution by labelling with barcoded RCA products deposited in wells 0.5 μm apart [59, 60]; *Stereo-seq* and other very high resolution sequencing techniques like *PIXEL-seq* [61] achieve similar mRNA recovery rates per unit area to *Visium* [17]. *Stereo-seq* is currently undergoing commercial development by BGI as its *STOmics* platform, currently in early access. Finally, while most of these techniques are designed for *fresh frozen* tissues stored below the temperature at which mRNAs degrade, some methods such as *Visium FFPE* are compatible with tissues that are fixed with formalin and embedded in paraffin wax, although this requires extra steps to prepare the tissue for profiling and a different, gene-specific probe-set (although all genes in the genome are nonetheless profiled). Independently developed techniques to adapt *Visium* reagents to FFPE-preserved tissues are also available but it is unclear whether these are in active commercial development [62].

An alternative to mounting tissue onto an array is to ‘print’ the array onto tissue using microfluidic channels, an approach used by *deterministic barcoding in tissue for spatial -omics sequencing (DBiT-seq)* [63]. Barcodes are deposited along one axis of a tissue section and then perpendicular to the first axis. The barcodes are then ligated to each other to produce a unique barcode at every point on the tissue. Capture area depends on the diameter of the microfluidic channel used, which can vary from 10 μm to 25 μm or 50 μm. This approach can be advantageous in avoiding potential diffusion of mRNA away from local capture areas and in allowing protein assessment by administering oligonucleotide-tagged antibodies in microfluidic channels prior to processing.

A disadvantage of these methods is that capture areas do not follow the complex contours of cellular morphology. Hence, cells often straddle multiple capture areas, contributing mRNA to more than one pixel. Even when capture areas are smaller than a single cell (as in *HDST*), they still lack single-cell resolution, since they capture mRNA merely from a single-cell-*sized* area. Recent techniques such as *XYZeq* [64] and *sci-Space* [65] have therefore employed a spatially-barcoded array not for mRNA capture but for intact cell labelling. Intact cells are then dissociated and undergo scRNA-seq with the spatially-recorded barcode. mRNA recovery in these methods benefits from using established scRNA-seq technologies, with *sci-Space* detecting a mean of ~ 1200 genes/cell. However, they operate at comparatively low spatial resolution. *sci-Space* uses 80 μm-radius spots and *XYZeq* spots have a centre-to-centre distance of 500 μm.

Array-methods have various advantages and disadvantages compared to ISH and ISS-methods. They often profile larger tissue sections, for example up to 6.5 mm × 6.5 mm for *Visium* compared to 0.5mm^2^ for *seqFISH*. By using NGS rather than microscopy, they avoid image-processing pipelines. Also, they are untargeted and can profile the whole transcriptome for any organism that uses polyadenylated mRNA. However, their spatial resolution and mRNA recovery rates are lower than ISH and ISS-methods [17]. Finally, by relying on a fixed array, transcripts from different cells can be captured at the same spot, meaning that sophisticated analyses are needed to determine what cell types were present at each spot.

### Other -omics

There are now techniques for performing spatial genomic and proteomic experiments. In association with transcriptomics, these can complement each other, allowing researchers to trace gene activity from epigenomic regulation, to transcription, and finally to translated protein. Spatial genomics has benefitted from advances in large scale smFISH-based technologies which can easily be adapted from targeting mRNA to targeting genomic DNA. For example, *DNA seqFISH+* [66] can reportedly target ~ 3000 chromosomal loci at a range of resolutions, from 25-kb resolution for a limited number of regions up to 1-Mb resolution for the whole genome. Imaging locations of specific loci allows the study of genomic organization across thousands of cells. The authors also demonstrate simultaneous multimodal profiling of mRNA and sequential immunofluorescence for nuclear structures such as lamina, speckle, nucleolus, and others. Likewise, ISS-methods such as *ReadCoor* (i.e. *FISSEQ*) have also been adapted to genomics as *in situ genome sequencing*, or *IGS*, for studying genome structure in 3D [26]. In this case, the 30 bp reads yielded by ISS are unsuitable for studying the genome, which consists of 3 billion bases and contains numerous repetitive regions. So, the authors inserted sequencing primers into the genome every 100–600 bp with Tn5 transposase, barcode insertion and RCA for each 100–600 bp region, *in situ sequencing* of barcodes, and finally *ex situ sequencing* (NGS) of each barcoded 100–600 bp region to generate a 150-bp read. This allowed each region to be traced to a region of the genome and based on its barcode also to a spatial area inside a nucleus. Finally, while both the techniques discussed here use imaging techniques, sequencing-based *DBiT-seq’s* approach of printing barcodes onto tissue prior to NGS can also be used to deliver barcodes for spatial labelling of genomic DNA after Tn5 transposition, allowing spatial profiling of chromatin accessibility (spatial *ATAC-seq* or *assay for transposase-accessible chromatin*) [27]. This technology is undergoing commercialization by the company AtlasXomics. Overall, there is now a suite of published techniques for studying genome structure and accessibility in spatial context, and we anticipate that these techniques will soon become commercially available.

Spatial *proteomics* offers direct measurements of protein localization and abundance in space. While transcripts are relatively easily profiled, profiling all proteins in a cell or tissue is much more challenging. In fact, the term ‘proteomics’ is perhaps misleading since the entire repertoire of proteins cannot yet be assessed. Nevertheless, some approaches have been devised, with broad approaches such as mass spectrometry analysis of fractionated organelles to identify enriched proteins, affinity-purification mass spectrometry to identify interactions by profiling proteins bound to their partners, and imaging-based proteomics [67]. Here, we briefly discuss antibody-dependent methods as an alternative to spatial transcriptomics, although it should be noted that reliance on validated antibodies means that only a fraction of the proteome can currently be assessed in organisms such as humans and experimental mice. Immunofluorescence is limited by the number of fluorophores that can be distinguished, so recent techniques such as *t-CycIF* and *CODEX* have used sequential methods to read out a barcode for an antibody [24, 25]; *CODEX* reads out antibody-conjugated DNA barcodes with fluorescent, hybridizing nucleotides, demonstrated with a 30-antibody panel. Mass cytometry uses antibodies labelled with metals, which are profiled with cytometry by time-of-flight. This is advantageous because it avoids problems of tissue autofluorescence and allows detection of all molecular targets at once. A recent method, multiplexed ion beam imaging by time of flight (*MIBI-TOF*), and imaging mass cytometry (*IMC*) have resolutions of 1 μm or less when profiling 36–40 proteins [23, 68]. Spatial proteomics techniques are increasingly being applied in research, such as in a recent study to characterize differential spatial activation and migration of macrophages with matrix-assisted laser desorption/ionization mass spectrometry imaging (MALDI-MSI) [69]. Overall, we anticipate that spatial proteomics will grow in prominence, although it does not yet yield genome-scale information.

## A guide to designing a spatial transcriptomic experiment

**Fig. 2:**
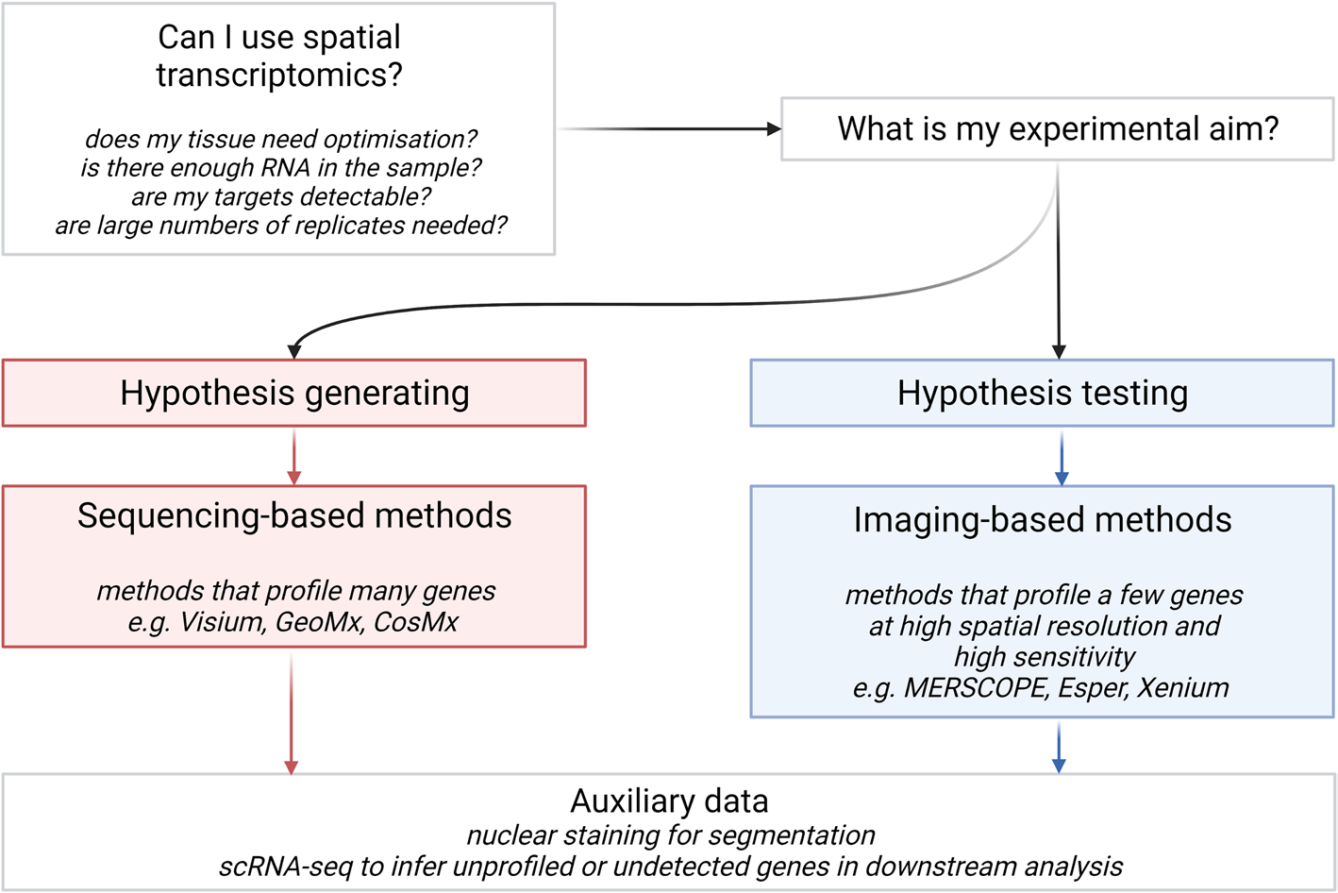
Design considerations for spatial transcriptomics experiments. Spatial transcriptomics technologies can satisfy a variety of experimental aims if the correct platform and design are chosen. Here, we have outlined a simple distinction between *hypothesis testing*—highly targeted experiments to examine regulation of defined genes and pathways—and *hypothesis generation*, which aims to reveal mechanisms without bias. Thus, we suggest hypothesis testing is best suited to efficient, targeted, and spatially-resolved ISH- and ISS-based methods. Conversely, hypothesis generation is best served by unbiased array- and microdissection-based methods that generate large volumes of data. However, researchers should note that there are a range of other considerations like the tissue type, quality of mRNA in the tissue, and amenability to generating a single-cell reference dataset that will also affect the choice of method or design

We have so far reviewed various spatial transcriptomics technologies, each with different strategies for capturing transcriptomes and preserving spatial information. These exhibit different technical capabilities, such as the size of the area profiled, number of genes that can be profiled, spatial resolution, and mRNA capture rate. Below, we review and frame these capabilities in the form of a guide for those considering their first spatial transcriptomic experiment (Fig. 2). Firstly, we discuss biological models and tissues amenable to spatial transcriptomics. Secondly, we discuss the practicality of different spatial transcriptomics techniques for different experimental aims. Thirdly, we discuss elements of experimental design such as number of samples, controls, and other considerations like paired histological imaging, paired protein detection, and matched single-cell RNA-seq references. Finally, we focus on a handful of techniques that are commercially available or nearing availability at the time of writing, representing the four classes of spatial transcriptomics techniques identified above: *MERSCOPE* (based on *MERFISH* ISH technology [45]), *Esper* (ISH-based) [46], *Xenium* (based on Cartana [49] and *FISSEQ* [50] ISS technologies), *Visium* (based on *spatial transcriptomics* array technology [20]), *STomics* (array-based) [60], *GeoMx* (microdissection-based) [21], and *CosMx* (ISH-based) [22].

### Can I use spatial transcriptomics?

Any intact tissue containing viable mRNA is suitable for spatial transcriptomics. As we have demonstrated, spatial transcriptomics techniques are useful in a variety of biomedical science subdisciplines such as neuroscience, cancer, immunology, and developmental biology. They also suit a variety of experimental designs ranging from atlas generation, untargeted hypothesis generation, and hypothesis testing. However, some initial considerations about the suitability of different tissues and models for different techniques must be made.

First, different tissues have different properties and spatial transcriptomics techniques must in some cases be *optimized*—that is, tailored—to a particular tissue. An advantage of established technologies such as *Visium* is the extensive list of optimized tissues covering most systems in human and mouse, as well as select tissues from rat and zebrafish. Optimization might aim to determine, for example, the optimal duration of tissue permeabilization for mRNA release. In *Visium*, optimization requires extra reagents and hands-on time but does not require more sequencing. Instead, conversion of mRNA to cDNA takes place on a *Visium* slide and cDNAs are then fluorescently labelled and imaged after a range of different permeabilization times to determine which time releases the most cDNA with the least lateral diffusion. Optimization is not a consideration for some methods like *Visium FFPE.* It remains to be seen whether other technologies nearing commercial release will require optimization. In addition to optimization, human brain tissues may exhibit autofluorescence due to lipofuscin, an age-related lysosomal residue in neurons which has a detrimental effect for imaging-based techniques [46, 70]. Some commercial methods may require extra processing steps to reduce autofluorescence.

Second, some structures and genes might be difficult to detect with spatial transcriptomics. For example, rare cells will be difficult to study via methods like *Visium* where a cell cannot be profiled individually but its transcriptome is mixed with surrounding cells. Small structures consisting of only a handful of cells could also pose a challenge. Finally, some genes such as transcription factors may be less transcribed than others, even though their function might have profound effects. Thus, methods with low capture efficacy might be ill-suited for studying spatial patterns of expression for lowly transcribed genes. Overall, we recommend that if studying rare features, researchers carefully select a suitable technique and perform experiments such as immunohistochemistry in parallel tissue sections to ensure the presence of the desired feature.

Third, the quality of tissue may affect the choice of whether to employ spatial transcriptomics. Previously, few techniques were capable of profiling preserved tissues such as formalin-fixed, paraffin-embedded tissues, as opposed to fresh-frozen tissues stored at low temperature to prevent mRNA degradation. However, this is no longer a limitation for many technologies*.* A more important consideration now is mRNA *quality* in preserved tissue. Over time, RNA degrades and fragments. A common measure of fragmentation is DV200—the proportion of RNA fragments over 200 nucleotides in length. For *Visium FFPE*, a DV200 of ≥ 50% is recommended. While this often correlates with the age of a tissue block, suboptimal storage can also accelerate mRNA degradation and DV200 testing is recommended to determine sample suitability. For degraded (DV200 < 50%) FFPE tissues or delicate fresh tissues, one replicate may be insufficient, particularly for array-based methods. This is because low DV200 may result in poor sequencing output. Furthermore, all commercial or near-commercial spatial transcriptomics methods use tissue sections, which are susceptible to tearing and distortion when placing on a slide or array for profiling. Incorporating replicates may improve the likelihood that a reliable sample is obtained. For some methods, researchers can easily obtain (although at a cost) more replicates by placing more tissue sections on a slide; for example, a *Visium* slide contains four separate arrays for profiling four different tissue sections simultaneously.

Fourth, the objective, design, and models employed in the experiment are paramount. If the objective is hypothesis testing—*e.g*. determining the spatial expression patterns of a handful of target genes or pathways in high resolution—then a targeted, high-resolution method is required, such as imaging-based spatial transcriptomics or perhaps spatial proteomics. Conversely, if the aim is hypothesis generation—examining unbiased, whole transcriptomes—then an untargeted method is preferred. Experimental design also affects whether spatial transcriptomics is appropriate. For example, if many samples are desired, an imaging-based spatial transcriptomics technique might be impractical due to the amount of imaging time required. Published ISH techniques, while efficient at detecting mRNA and spatially highly resolved, can take weeks to image a large dataset [46, 71]. While we anticipate that techniques like *Esper* will have lower imaging time with no user input required, it will still be an obstacle for experiments with multiple samples to be imaged consecutively. Likewise, while sample preparation can be parallelized for sequencing methods like *Visium*, costs will accrue with many samples not only for spatial transcriptomic reagents but also for NGS and for generating companion scRNA-seq datasets (discussed below). Therefore, spatial transcriptomics techniques are currently not suited to experiments involving many samples, for example longitudinal assessments of tissues from multiple experimental animals. Because of this limitation, we suggest that spatial transcriptomics is best applied either to well-characterized, reproducible experimental systems, or to demonstrably representative samples where human tissue is used. For example, a parallel tissue section could be taken from the tissue of interest and profiled with immunohistochemistry to determine its suitability for spatial transcriptomics.

Having considered, tissue type, sample integrity and experimental objectives, multiple technical parameters, outlined below, should be considered:

- *Sample number*: For ISH and ISS-methods, e.g. *MERSCOPE*, *Esper*, *Xenium*, each sample is individually, repeatedly imaged using a specialist instrument, meaning that only one sample is generally assessed at a time. In array-methods, multiple sections can be assessed on one array, e.g. 4 sections per slide for *Visium.* (All methods discussed here used comparable tissue section dimensions, > 1 cm × 1 cm)
- *mRNA capture efficiency*: ISH-methods, e.g. *MERSCOPE* and *Esper*, typically capture more of the available target mRNAs than ISS-based methods or sequencing-based methods. Efficiency can range from nearly 100% in ISH-based methods to as low as 1–2% for some array-based methods
- *Spatial resolution*: ISH and ISS-methods can achieve subcellular resolution. In sequencing-based methods, mRNAs are released and collected on an array of fixed spatial resolution or in an ROI larger than a single cell. To date, only *STomics* achieves comparable resolution to ISH and ISS-methods (< 1 μm)
- *Number of genes profiled*: *MERSCOPE* profiles up to 1000 genes, and *Esper* up to 5000, array-based methods are untargeted, therefore providing genome-scale coverage

Based on these factors, we suggest that hypothesis testing experiments are best suited to ISH- and ISS-methods like *MERSCOPE*, *Esper*, and *Xenium.* An untargeted but high-resolution technology such as *CosMx* may also be suitable. For example, a validation experiment to confirm differential gene regulation relative to a spatial feature does not require whole-transcriptome profiling but would benefit from the increased spatial resolution and mRNA capture efficiency of ISH- and ISS-based methods. Conversely, unbiased hypothesis generation and atlas generation experiments, perhaps with large tissue areas, are best suited to array-based methods like *Visium* and *STOmics.* For example, a discovery experiment aiming to uncover new pathways in a previously uncharacterized tissue would benefit from unbiased, whole-transcriptome profiling.

### What instruments are needed?

All spatial transcriptomics techniques require instrumentation. For ISH and ISS-methods, the primary instrument will be an imager, whereas array methods require an NGS sequencing platform. *MERSCOPE* and *Esper* provide bespoke imaging instrumentation (presumably also the case for unreleased *Xenium*) and software to handle preprocessing steps for image analysis (discussed below). Some sequencing-based methods require instruments for tailored mRNA capture—e.g. *GeoMx* and *CosMx*—whereas *Visium* requires no specific instrumentation at all except access to NGS. While many of the techniques discussed here are in active commercial development, not all of them are yet available, so comparative capabilities and costs of each instrument are not clear.

### Incorporating scRNA-seq and staining

Designing a spatial transcriptomic experiment requires careful consideration of technical and experimental parameters to ensure that aims are met. However, in this section, we will discuss some other data types that increase the utility of a spatial experiment.

Most spatial transcriptomics methods exhibit either low mRNA detection efficiency—around 10% or less of mRNAs for array-based methods—or smaller targeted gene panels. For example, *MERSCOPE*, profiles up to 500 genes or about 2.5% of the genome (albeit at high sensitivity). Furthermore, array-based methods use fixed mRNA capture areas and therefore do not have *cellular* resolution, even if the capture area is smaller than a cell, because mRNA may derive from multiple cells overlapping the capture area. Even for imaging-based methods with single mRNA resolution, single-cell transcriptomes must usually be computationally reconstructed during analysis [17]. Therefore, many spatial techniques are not truly ‘single cell’ and can benefit from a companion single-cell RNA sequencing dataset from the same tissue. For targeted technologies, an unbiased single-cell reference can be used to infer expression of genes that were not measured spatially, a task accomplished by numerous published bioinformatic tools, or for assigning individually imaged mRNAs to single cells. For array-based methods, a single-cell reference is often used to infer what cell types, and in what proportions, contributed to the mixture of mRNA in each capture area; this process is called *deconvolution*. Second, in some cases, the reference may be used to impute expression of genes that were poorly profiled by spatial technology. Overall, when designing a spatial transcriptomic experiment, we recommend that researchers working with tissues amenable to dissociation consider generating a reference single-cell RNA-sequencing dataset. This may not be possible for delicate tissues such as human brain.

Next, we recommend that researchers consider whether their selected spatial transcriptomics technique is compatible with auxiliary tissue staining. Staining, for example for nuclei, is advantageous for techniques with subcellular resolution such as imaging-based methods. In these methods, the locations of individual mRNAs are recorded, but the locations and extent of whole cells are not. Staining for nuclei, for example with DAPI, or for cell boundaries can help to infer the *locations* of cells and to computationally reconstruct single-cell transcriptomes from the observed mRNAs. We discuss methods for computationally identifying single-cell transcriptomes from data with subcellular resolution, or *segmentation*, below. Furthermore, some bioinformatic techniques such as *stLearn* can leverage stain imaging to identify tissue domains and features in tandem with gene expression data from sequencing-based techniques. Currently, only *Visium* and *GeoMx* offer paired auxiliary staining, e.g. H&E, among sequencing-based techniques.

## A guide to spatial transcriptomic analyses

**Fig. 3:**
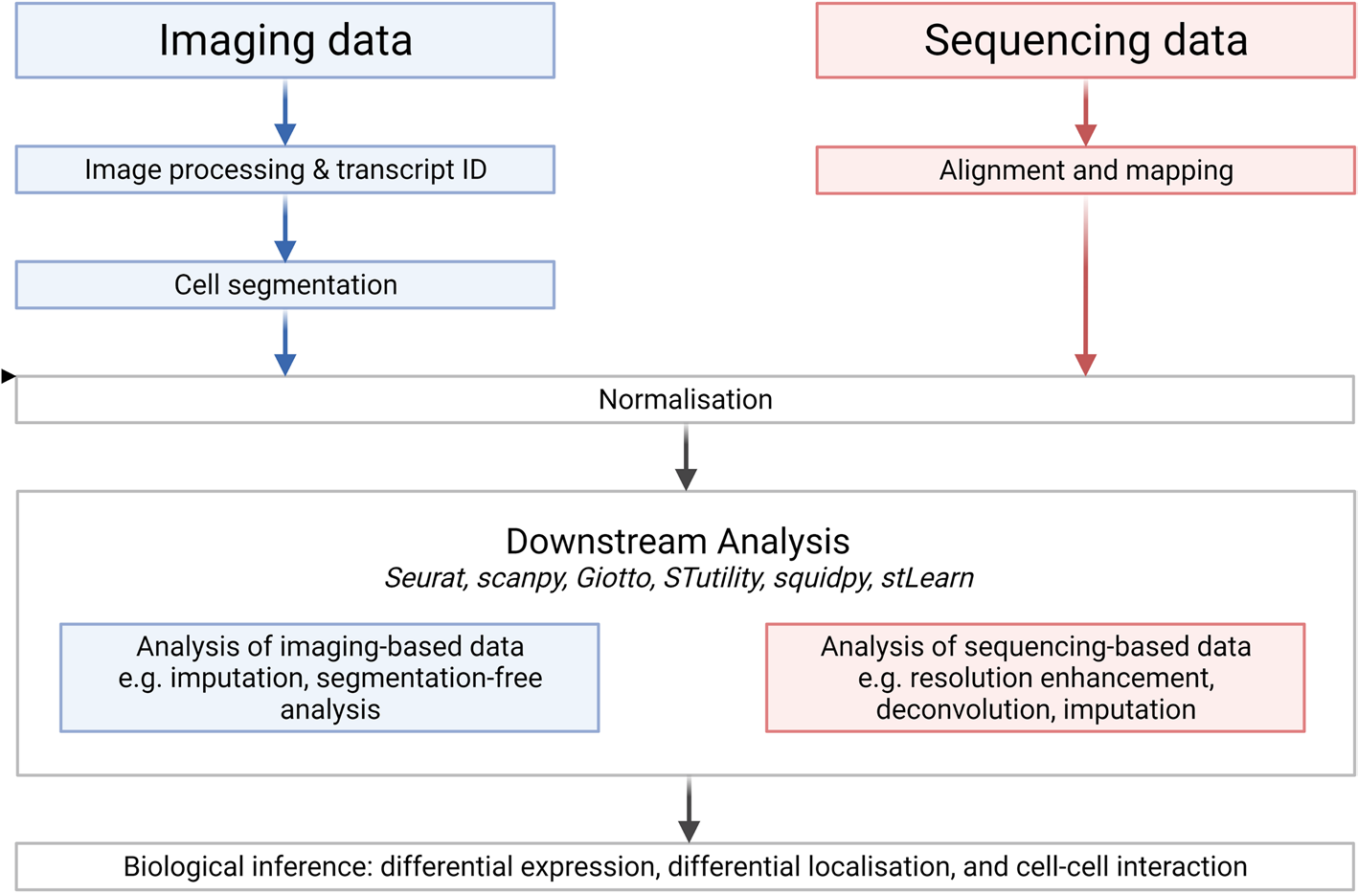
Typical structure of spatial transcriptomics analysis. Data are first preprocessed using technique-specific methods and algorithms. Normalization methods account for technical variation. Downstream analyses may be performed with a range of general-purpose transcriptomic analysis packages or with specialized methods for spatial transcriptomics. Created with biorender.com

Techniques for analysing spatial transcriptomic data have proliferated over recent years. They serve a variety of purposes, including preparing, or ‘pre-processing’ data for analysis (e.g. aligning microscopy images of labelled mRNAs), conducting biological analyses (e.g. spatial differential gene expression), combining, or ‘integrating’ with scRNA-seq data, or inference of cell-cell interactions (Fig. 3). Below, we review pre-processing steps and provide guidance on some common analyses, with recent examples of each.

### Pre-processing spatial transcriptomic data

One of the first analytical tasks to perform is to prepare spatial transcriptomic data for analysis, known as *pre-processing*. This usually serves to convert raw imaging or sequencing data to a matrix of *transcript counts per gene* by *spatial capture areas*, which we will refer to as *gene-spot matrices* (in multi-omic methods, genes may be accompanied by protein counts), with specific methods required for data generated by different techniques (Fig. 3)*.*

Typical ISH- and ISS-based methods will read out a sequence or gene-specific barcode over multiple hybridization rounds, so the images are uninformative on their own. There are several steps required to convert these images to a gene-spot matrix. First, images are filtered to remove background and noise. Second, images from different hybridization rounds are *aligned* so that the same pixel location or *spot* in each one represents the same transcript. Third, signals at each spot are combined into a barcode or sequence that can be used to match spots to genes, with signals that do not match any gene rejected. Finally, an optional step is *segmentation*, discussed below*.* Tools for pre-processing ISH or ISS-based transcriptomics are often targeted to a particular technique, e.g. *MERFISH* or *seqFISH*, but the recent package *starfish* provides a well-documented and more general-purpose pipeline [17, 72]. Like 10X Genomics’s pre-processing pipelines for data generated via their single cell and spatial transcriptomics platforms, we expect that imaging-based platforms such as *MERSCOPE*, *Esper*, and *Xenium* will soon receive dedicated pre-processing pipelines.

For *Visium*, the only commercially available array-based method, 10X Genomics has published a pre-processing pipeline—*Space Ranger*—that performs pre-processing with minimal user input. To convert sequencing data to spatial transcriptomic data, it accepts raw sequences of captured mRNAs and microscopy images of the profiled tissue, performs alignment of reads to the genome, matches read barcodes to spatial locations in the array, and counts the number of gene transcripts at each spatial location to produce a gene-spot matrix.

A final pre-processing step in some methods is *segmentation*. The aim of segmentation is to reconstruct single-cell transcriptomes from spatial data with subcellular resolution. For example, segmentation could be used with imaging-based data from methods such as *MERSCOPE*, *Esper*, or *Xenium* to reconstruct single-cell transcriptomes by inferring from transcript species and clustering which areas of the image likely encompassed one cell. Similar approaches can be used with subcellular array-based data, e.g. *STOmics*, which generates sub-micron resolution data. Thus, segmentation transforms a gene-spot matrix into an inferred gene-cell matrix. There are numerous published segmentation methods using different approaches such as manual segmentation, prior information from nuclear staining [73], deep neural networks [74], gene expression signatures from true single-cell references generated by scRNA-seq [73, 75], and some workflows such as *spot-based spatial cell type analysis by multidimensional mRNA density estimation* (*SSAM*) avoid segmentation entirely [17, 76]. Perhaps one reason for the diversity of approaches is that cell segmentation is a complex and computationally expensive process, especially when many cell types are present [73]. Where segmentation is required, we recommend approaches that can leverage prior information from nuclear staining and scRNA-seq references, such as *Baysor* [73]. As with other pre-processing steps, we anticipate that commercial platforms such as *MERSCOPE*, *Esper*, and *Xenium* will ship with built-in segmentation pipelines.

One final step in pre-processing the data is to apply statistical transformation to the gene-spot matrix to account for differences in mRNA capture rate across the tissue. This is an important step for data generated via all techniques but especially those with lower or variable capture rates such as array-based methods. In scRNA-seq, the process of accounting for differences in mRNA capture between dissociated cells is called *normalization*, and the same terminology is applied in spatial transcriptomics. The most common normalization procedure is to divide each cell in a gene-spot matrix by the spot total, so that every spot has the same number of counted mRNAs in the processed matrix. This approach is used by general-purpose analysis packages such as *Scanpy*, *Giotto*, and *Seurat*. However, this approach assumes that all regions of the tissue have the same underlying mRNA abundance, or ‘library size’, an assumption that may not be true for tissues with regions of dense nuclei juxtaposed with regions of sparse nuclei and thus lower mRNA abundance. *Seurat* offers the method *sctransform*, which normalizes not by total library size of each cell’s transcriptome but based on one group of genes at a time, with each group selected so that all the genes have similar abundances. Thus, the approach allows variation in total library size rather than enforcing it as a constant metric. This is likely to be favourable for tissues with underlying variations in mRNA abundance driven by differential cell density. Also, in contrast to standard normalization, *spatial and morphological expression* (*SME*) from the *stLearn* python package presents a novel method for normalization which smooths library sizes in spots or segmented cells based on the library size of nearby spots within a radius *d* and their morphological similarity inferred by deep learning of features from histological images while preserving larger-scale variations in mRNA abundance across the tissue [77]. Finally, one report suggests that unnormalized (‘raw’) data are also informative and preserve information about cell densities [78], but their suitability for downstream analyses is not assessed so we recommend either library size normalization or specialized techniques such as *sctransform* and *SME* depending on the library size variation across the sample.

Overall, several steps are required to convert raw image or sequencing data to processed, interpretable spatial transcriptomic data. The steps required vary between technologies, but there are tools to handle each of them, often published as a complete pre-processing pipeline such as in *starfish* for imaging-based data or *Space Ranger* for *Visium* data, as well as other method-specific pipelines that we anticipate will be released on instrument computers as in *MERSCOPE*. The final step mentioned above, normalization, is often handled by separate pipelines for *downstream analysis*, a broad term encompassing all analysis techniques that aim to generate or test biological hypotheses with the data. Below, we will review some common pipelines for downstream analysis and examples of analyses specific to spatial data.

### Generalized toolkits for downstream analysis

There are a range of different downstream analyses for spatial data, with different aims and different inputs. Spatial data may comprise raw gene-spot matrices, normalized matrices, or accessory data such as inferred cell types and tissue domains to histological images taken before transcriptomic profiling. To provide a unified format for these data, and to simplify and standardise spatial analysis, utility packages such as *Giotto*, *STUtility*, *Seurat*, *scanpy*, *stLearn*, and *squidpy* have been developed [77, 79–83]. Among their shared aims are, first, to provide a structure for spatial data matrices and for associated accessory data generated through downstream analysis. The latter might include dimensionality reductions such as UMAP, unbiased clustering results, annotations, and imputation, mapping or deconvolution results. A second aim is to provide functions to complete all those processes. Third, they provide functions for data visualization, combining spatial transcriptomic data with overlays such as microscopy data [81, 83]. Finally, they provide standardized workflows for quality control (e.g. filtering poorly expressed genes), pre-processing, and specialized analysis techniques for spatial data.

For a researcher selecting an analysis package, they most obviously differ in terms of capabilities, size of user communities, and the uptake of their data formats in the larger bioinformatics community. Currently, *Seurat* (in R and cited > 4700 times at the time of writing) and *scanpy* (in python and cited > 1600 times at the time of writing) benefit from extensive documentation generated over years, from large user communities, and from many packages that recognize or even operate directly on their formats (*SeuratObject* and *anndata*, respectively). Conversely, *Giotto* (in R) and *stLearn* (in python) benefit from workflows developed specially for spatial transcriptomics and a greater variety of built-in tools for spatial downstream analyses. These include spatially variable gene identification, deconvolution, and cell-cell interaction inference, all outlined in the following section. Finally, *STUtility* and *squidpy* provide extended spatial analysis functions for *Seurat* and *scanpy*, respectively. *STUtility* focusses on analysis of multiple spatial transcriptomic datasets and contains features for annotating tissue regions, alignment of parallel 2-dimensional spatial datasets, and visualization of resulting 3-dimensional datasets. *Squidpy* likewise extends *Scanpy* and is from the same authors but provides a depth of functionality akin to *Giotto* with specialized data structures, tools for performing spatial statistics, inferring intercellular interactions, and visualizing data. Finally, *STUtility*, *squidpy*, and *stLearn* provide functions for analysing auxiliary image data. This can be challenging as the images are large and require significant memory to work with. *STUtility* works with H&E images generated alongside Visium data and transforms and aligns low resolution versions of these images before applying the transformations to higher resolution images; it uses these data for alignment of sequential sections and regional annotation. In contrast, *squidpy* provides a format for image data, *ImageContainer*, with lazy loading rather than reduced resolution to conserve memory, as well as a suite of analyses to make use of these data. Finally, *stLearn* provides functions for incorporating image data with gene expression data such as in the normalization step (discussed in the previous section). Overall, we recommend *Seurat* and *Scanpy* for lay biomedical researchers due to their extensive documentation and large user communities and recommend *Giotto*, *stLearn*, *STUtility*, and *squidpy* for researchers seeking more specialized spatial transcriptomics analysis pipelines.

### Identification of spatial features

An initial goal for many scRNA-seq analyses is to define the cell types. An analogous step in spatial transcriptomics analysis might be the identification of spatial features such as anatomical and microanatomical structures. To identify structures, existing algorithms can group transcriptomically similar spots or cells in an unbiased way to reveal spatial patterns of gene expression. However, newer methods have been developed to specifically leverage spatial data and identify features like tissue domains. Examples include *BayesSpace*, a Bayesian statistical tool that uses a clustering approach, albeit with prior spatial information, to group transcriptomically similar, proximally located spots. Additionally, it can perform resolution enhancement by reassigning gene counts from whole spots in array-based data, e.g. *Visium*, to finer sub-spots with the use of spatial prior information from nearby spots [84]. Similarly, *XFuse* combines auxiliary histology (usually H&E) images with gene expression data via deep data fusion of spatial features to infer gene expression *between* spots in an array [85]. Other algorithms for tissue domain identification include *stLearn* [77], which uses *SME* normalization with inference from histology images via deep learning (see pre-processing methods above) to infer clusters and then subclusters where there is spatial segregation, and *HMRF* (hidden Markov random field) [86], which assigns spots or cells to tissue domains as a function of their gene expression, and the domain in which neighbouring cells reside, which is included in the *Giotto* analysis package. Overall, the choice of method will depend on the data available; for array-based methods with low resolution, *BayesSpace* might be favourable for resolution enhancement, but if histological images are available, then *stLearn* will be powerful for its ability to integrate them in its analysis of spatial transcriptomic data.

Alternatively, rather than identifying heterogeneity among cells and spots across the sample, one might search directly for genes that show biased, non-random spatial expression patterns. This can quickly elucidate anatomical features if known marker genes are detected. Numerous methods for detecting genes that vary spatially have emerged over the past few years, with some implemented in popular tools such as Seurat as the function *FindSpatiallyVariableGenes* which estimates spatial autocorrelation with Moran’s l over binned groups of spots rather than over individual spots for improved speed; in Giotto as *BinSpect-k* means or *BinSpect-rank*, both of which use (separate) techniques to binarize expression data and examine the correlation of a gene’s expression in one spot with that in neighbouring spots to estimate a *p* value; or as standalone analysis tools such as *trendsceek*, *SPARK*, and *SpatialDE* in python [81, 86–89]. Notably, Giotto’s methods for spatially variable gene selection provide improvements in speed over some older methods such as *SpatialDE*, *trendsceek*, and *SPARK*, a key concern given the continuing trend of larger datasets in spatial transcriptomics [79]. *sepal* is a more recent method which takes a novel approach, simulating the time taken for observed transcripts of a single species to diffuse across the sample to a random distribution, with this metric inferring the degree of spatial structure underlying the species’ distribution [90]. Binning approaches may be used to improve speed but this could result in loss of spatial detail depending on the size of the bins used.

### Deconvolution

An important consideration for data generated by array methods with non-cellular resolution is that more than one cell type can contribute to each spot. This is particularly important for *Visium*, since its spatial resolution of 55 μm means each spot may capture many cells, the exact number depending on the tissue. The process of identifying and quantifying the relative contribution from each cell type in a capture spot is known as *deconvolution.* Numerous tools have been developed to perform this task, usually from an scRNA-seq reference. An early example was *NMFreg*, an approach that decomposed spot transcriptomes into contributions from cell types by non-negative matrix factorization, developed for deconvolution of *Slide-seq* (V1) data [56]. *SPOTlight* also utilizes non-negative matrix factorization to estimate cell type proportions [91]. This was followed by robust cell type decomposition (*RCTD*), which used a different statistical model to explain gene counts in each spot as a mixture of cell type contributions, unobserved platform—e.g. single-cell or spatial transcriptomics—effects, and spot-specific mRNA sampling effects, allowing it confidently assign cell types to spots much more frequently than in *NMFreg* (86.9% of spots vs 24.8%) [92]. Because of the similar data structures between *Slide-seqV2* and *Visium* data, these methods are also applicable to the latter. Alternatives include *stereoscope*, *cell2location*, *Tangram*, and *destVI* [93–96]. *stereoscope*, like *RCTD*, models the composition of each spot’s transcriptome as a mixture of transcripts from different cells with additional platform-specific effects. *Cell2location* and *destVI* are both contained within the *scVI* analysis framework and use deep learning approaches to achieve relatively high speed, as does *Tangram*. *destVI* is unique in that compared to the other tools discussed, which deconvolute spatial data from a reference of discrete cell types, it maps continuous cell types. In effect, this allows it to map not only a known reference cell type but also variation within that cell type. *Tangram* also incorporates imaging data such as H&E staining during its deep learning process to first segment cells in the image and to use this as the basis for the number of cells inferred through deconvolution. Finally, utility packages such as *Seurat* and *Giotto* provide deconvolution methods [79, 81]. Giotto’s methods, *PAGE* and *RANK*, perform comparably in accuracy to *RCTD* [79]. Thus, to deconvolute spatial transcriptomic data not of single-cell resolution requires access to a ‘ground truth’ scRNA-seq dataset. When selecting a deconvolution technique, we suggest that users consider the run time as this step can require significant computing time and power. Recent benchmarking studies will also help users select an algorithm [97].

### Imputation and mapping single cells

Some computational methods aim to combine spatial transcriptomic and scRNA-seq, not for reasons of deconvolution, but to infer where genes, while not detected, may actually have been expressed. This allows users to ‘fill in the blanks’ where spatial transcriptomic data’s targeted nature or, for some approaches, low sensitivity means a gene is not detected, a task known as *imputation*. Conversely, some methods take the opposite approach, using spatial datasets to infer spatial mappings for scRNA-seq-derived single-cell transcriptomes, for example in Visium where single-cell measurements cannot be made.

An early integration approach was *Seurat* (prior to becoming a general-purpose analysis package), which maps single-cell transcriptomes to spatial coordinates. *Seurat* maps cells by expression of ‘landmark genes’, inferring probabilities that a cell could have originated from a tissue location whose landmark genes match its own [98, 99]. Since then, multiple other computational approaches have been devised. More recent mapping approaches include *SpaOTsc*, which relies on an optimal transport model to map single-cell transcriptomes to spatial data and also includes functions for inferring ligand-receptor interaction [100]. Imputing approaches include deep learning-based *gimVI* which learns an alignment between scRNA-seq and spatial transcriptome data, included in the python-implemented scRNA-seq analysis package *scVI*; *Tangram*, which uses a mapping step to inform the imputation process; and *spatial gene enhancement (SpaGE)*, which aligns spatial and scRNA-seq data by domain adaptation to inform imputation [96, 101, 102]. *gimVI* and *Tangram* are deep learning-based methods, so the choice of method may depend on whether GPU computing resources are available to researchers (they can be performed on CPUs, albeit with greater computing time). Mapping of single-cell transcriptomes will likely be useful for non-single-cell data such as unsegmented imaging-based data or data from any sequencing-based method. Conversely, imputation will be useful for inference of unmeasured genes in targeted spatial transcriptomic data.

### Cell-cell interaction inference

A common goal in analysing transcriptomic data is to infer intercellular interactions based upon expression of ligands and receptors [9]. Typically, a cell-cell interaction inference tool combines a database of genes encoding proteins proven to participate in intercellular interactions, with an algorithm to infer the probability of an interaction from the gene expression data. There are now several examples of these tools for scRNA-seq data including *CellPhoneDB v.2.0*, *iCellNet*, *CellChat*, and *SingleCellSignalR* [103–106]. While effective for single-cell and some spatial data, these tools cannot leverage spatial data when it is available. Recently, several techniques have been developed for this purpose including *SpaOTsc*, *cell2cell*, *MISTy*, and *CellPhoneDB v.3.0* and one implemented in the general-purpose spatial transcriptomics analysis package *Giotto* [28, 79, 100, 107–109]. Some of these techniques, such as *SpaOTsc*, can also infer differential gene expression with proximity to a signal-sending cell. More recent methods include graph neural network-based *NCEM*, or node-centric expression model, which takes as input segmented data from imaging-based spatial transcriptomics or proteomics and can be used to infer which cells are signal senders or receivers, as well as to infer domains in the tissue [110], and spatial variance component analysis or *SVCA*, which uses a Gaussian process-based framework to decompose gene expression variation across spots into intrinsic effects, environmental effects, and intercellular signalling effects [111]. We favour these recent tools for multipurpose analyses and in *SVCA* for its ability to model intrinsic gene expression perturbations, although users might also find built-in databases useful as in one author’s *CellPhoneDB v.3.0*.

A range of techniques are required to infer biological processes from spatial transcriptomics: technique-specific methods to prepare data, to identify regions corresponding to single cells and tissue regions from transcriptomes alone, to infer genes that may not have been profiled by spatial transcriptomics but instead by another modality, and to identify spatially-mediated biology such as cell-cell interaction. Here, we have reviewed examples of tools for common spatial transcriptomics analyses; while we have commented on where these tools might be useful, we anticipate that many more, possibly more advanced, tools will be published beyond the time of writing.

## Conclusions

Selecting a spatial transcriptomic method from those commercially available at the time of writing requires decision-making based on parameters such as spatial resolution, tissue area, mRNA detection sensitivity, and number of genes profiled. We have proposed a framework for selecting the method that best aligns with research objectives along with experimental design considerations like number of samples required and other data modalities that can complement spatial transcriptomics. Given the fast pace of technology development, we expect new methods will appear that combine their best aspects to provide the ideal technology, that of single-cell spatial resolution *and* genome-scale gene expression profiling at high sensitivity. In the meantime, improved multi-omics, auxiliary stains, and single-cell RNA-seq references will allow more powerful and flexible bioinformatic analyses. Recent advances in assaying FFPE tissues will also dramatically increase utility in clinical and biomedical research.

## Data Availability

Not applicable
